# Biochar Amendments Improve Licorice (*Glycyrrhiza uralensis* Fisch.) Growth and Nutrient Uptake under Salt Stress

**DOI:** 10.3390/plants10102135

**Published:** 2021-10-08

**Authors:** Dilfuza Egamberdieva, Hua Ma, Burak Alaylar, Zohreh Zoghi, Aida Kistaubayeva, Stephan Wirth, Sonoko Dorothea Bellingrath-Kimura

**Affiliations:** 1 Leibniz Centre for Agricultural Landscape Research (ZALF), 15374 Müncheberg, Germany; swirth@zalf.de (S.W.); belks@zalf.de (S.D.B.-K.); 2 Department of Biotechnology, Al-Farabi Kazakh National University, Almaty 050040, Kazakhstan; kistaubayeva.kaznu@gmail.com; 3 School of Life Sciences, Chongqing University, Chongqing 401331, China; 4 Department of Molecular Biology and Genetics, Faculty of Arts and Sciences, Agri Ibrahim Cecen University, Agri 04100, Turkey; balaylar@agri.edu.tr; 5 Faculty of Natural Resources and Marine Sciences, Tarbiat Modares University, Noor 46417-76489, Iran; zohre_zoghi@yahoo.com; 6 Faculty of Life Science, Humboldt University of Berlin, 14195 Berlin, Germany

**Keywords:** biochar, licorice, soil enzymes, salinity, nutrients, root system

## Abstract

Licorice (*Glycyrrhiza uralensis* Fisch.) is a salt and drought tolerant legume suitable for rehabilitating abandoned saline lands, especially in dry arid regions. We hypothesized that soil amended with maize-derived biochar might alleviate salt stress in licorice by improving its growth, nutrient acquisition, and root system adaptation. Experiments were designed to determine the effect of different biochar concentrations on licorice growth parameters, acquisition of C (carbon), nitrogen (N), and phosphorus (P) and on soil enzyme activities under saline and non-saline soil conditions. Pyrolysis char from maize (600 °C) was used at concentrations of 2% (B2), 4% (B4), and 6% (B6) for pot experiments. After 40 days, biochar improved the shoot and root biomass of licorice by 80 and 41% under saline soil conditions. However, B4 and B6 did not have a significant effect on shoot growth. Furthermore, increased nodule numbers of licorice grown at B4 amendment were observed under both non-saline and saline conditions. The root architectural traits, such as root length, surface area, project area, root volume, and nodulation traits, also significantly increased by biochar application at both B2 and B4. The concentrations of N and K in plant tissue increased under B2 and B4 amendments compared to the plants grown without biochar application. Moreover, the soil under saline conditions amended with biochar showed a positive effect on the activities of soil fluorescein diacetate hydrolase, proteases, and acid phosphomonoesterases. Overall, this study demonstrated the beneficial effects of maize-derived biochar on growth and nutrient uptake of licorice under saline soil conditions by improving nodule formation and root architecture, as well as soil enzyme activity.

## 1. Introduction

Licorice is a perennial shrub widely distributed in South-Western Asia and the Mediterranean region, which consists of about 20 species. The most commonly grown species are *Glycyrrhiza glabra* L. and *Glycyrrhiza uralensis* Fisch., used as food and in traditional medicine to treat various health disorders [1,2,3]. The plant is well adapted to saline and arid soils because of its deep root system. Thus, licorice is used to restore soil fertility by increasing soil organic matter and soil biological activity [4,5]. However, exposure of licorice to excessive salinity may elicit nutrient deficiency and imbalances of N, K, P, and microelements, which are essential for plant growth [6,7]. Furthermore, salt stress disturbs the symbiotic performance of legumes with *Rhizobia*, thereby resulting in decreased nodulation and nitrogen fixation [8]. This process was explained by the failure of rhizobial colonization in the rhizosphere, and the formation of root nodules. There are several approaches to improving plant growth and development under drought and salinity, such as genetic engineering and the application of microbial inoculants [9]. Biochar amendment has also been repeatedly reported as an effective approach in restoring saline lands and increasing plant tolerance to salt stress; thus, it has gained attention in practical applications worldwide [6,10,11]. Biochar is a solid by-product of biomass-derived from forestry, agriculture, and the food industry, such as wood chips, crop residues, sewage sludge, or dairy manure. It is produced by pyrolysis or high temperatures under limited or complete absence of oxygen [12]. The positive effects of biochar on soil cation exchange capacity [13], water holding capacity [14] or soil organic matter content [15] were repeatedly demonstrated. Biochar also increased organic carbon in saline soil, supporting favourable conditions for soil microbes involved in nutrient cycles [16]. Tang et al. [17] also observed biochar-induced improvements in soil physical-chemical properties, which mitigated salt stress during seedling growth of *Brassica chinensis* L. Moreover, the soil under saline conditions amended with biochar experienced increased enzymatic activities such as of urease, invertase and phosphatase [18].

Biochar also plays a vital role in plant growth, providing nutrients and better nutrient availability [19]. The improved acquisition of nutrients such as N, P, K from soil amended with biochar was explained by enhanced bioavailability and increased microbial activity involved in nutrient cycling [20,21,22]. However, plant growth, nutrient acquisition, and soil biogeochemical processes after biochar application depend on the type and concentration of biochar. For example, Ibrahim et al. [23] observed that soil amendment with 2.5% biochar alleviated the harmful effects of salt stress in Sorghum (*Sorghum bicolor* L. Moench), whereas 5 and 10% biochar application rates harmed the plant growth under saline conditions. Our study hypothesized that the application of biochar mitigates the salt stress of licorice, improves soil nutrient acquisition through improving soil biological properties and root growth, and finally, that any beneficial effects depend on biochar concentration. Thus, our study could expand knowledge about the impact of increasing biochar concentrations on the improvement in the licorice root system (growth and architecture), symbiotic performance and nutrient availability, especially in salt-affected soils. Experiments were conducted in a greenhouse and included measurements of soil enzyme activities linked to carbon, nitrogen and phosphorus cycling.

## 2. Results

### 2.1. Plant Shoot and Root Growth

**Figure 1:**
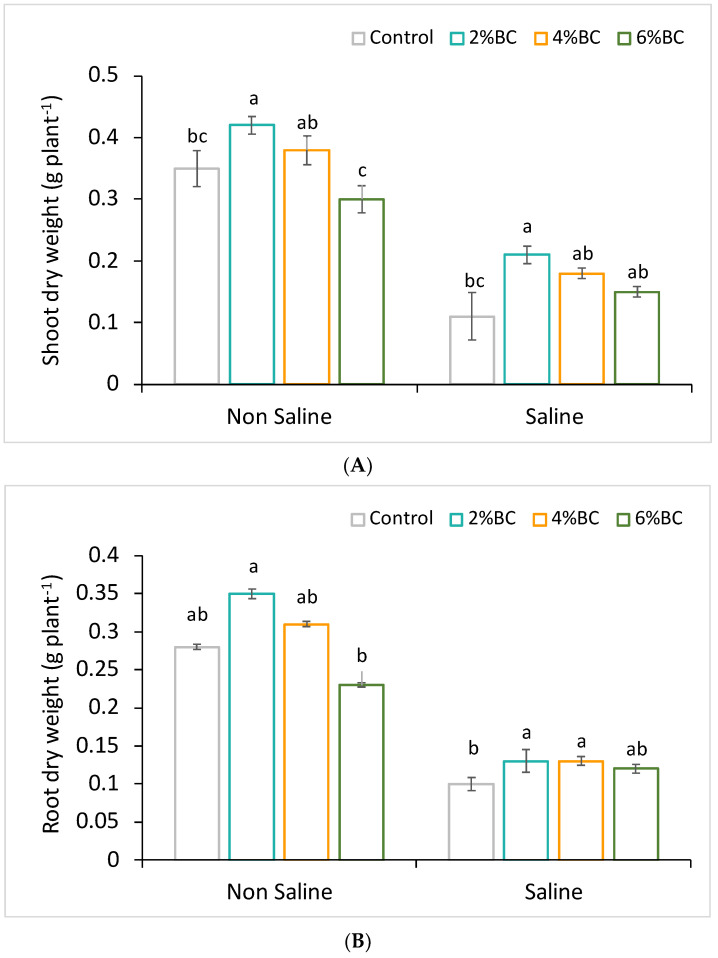
Shoot (**A**) and root (**B**) growth of licorice grown in soil amended with biochar at 2%, 4% and 6% concentrations under non-saline and saline soil conditions. Column means with different letters are significantly different based on Tukey’s HSD test at *p* < 0.05.

**Figure 2:**
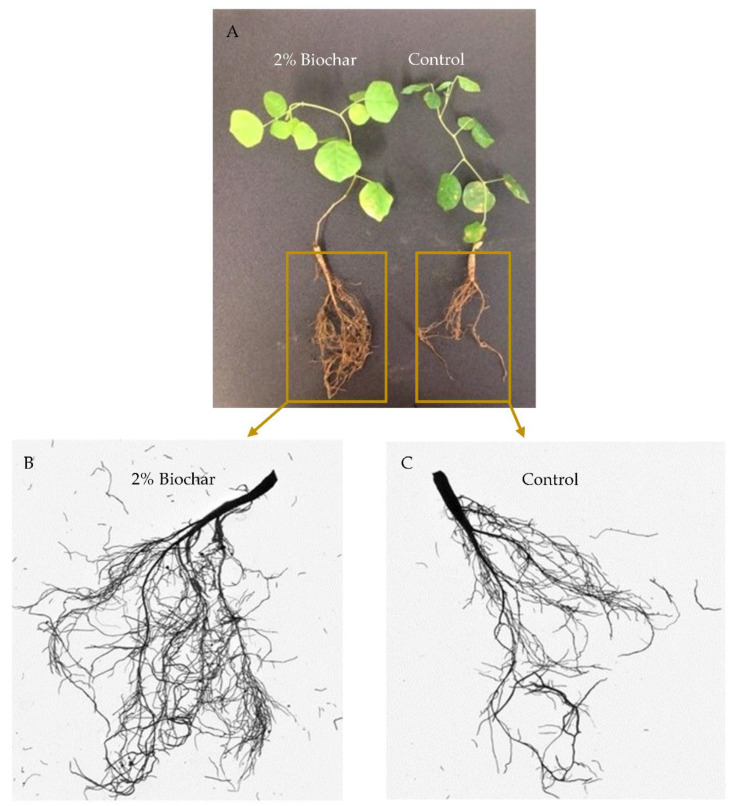
Biochar addition (2%) enhanced whole-plant growth under saline soil conditions. (**A**): entire plants, (**B**): amplified root system of biochar-treated variant, (**C**): amplified root system of control plant.

The licorice’s root and the shoot biomass responded differently to the applied biochar concentrations of B2, B4 and B6 and soil salt stress. In non-saline soil amended with B2, the shoot and root growth of licorice significantly (*p* < 0.05) increased by 25 and 28% compared to plants grown in soil without biochar addition (Figure 1 and Figure 2). However, there were no significant effects of B4 and B6 on licorice growth. The shoot and root biomass of plants even decreased after the amendment of B6 (Figure 1). Under saline soil conditions, biochar improved shoot and root biomass of licorice by 80 and 41%, respectively; however, B4 and B6 had no significant effects on shoot growth (Figure 1).

Notably, biochar improved the symbiotic performance of licorice, since the nodule number of plants grown in non-saline soil was 3.0 ± 1.0. In contrast, nodule numbers significantly increased threefold (8.6 ± 1.5) after the addition of B2 to the soil and twofold (6.0 ± 2.1) per plant at B4. Soil salinity inhibited nodule formation in the plant; no nodules were found on roots grown in saline soil without biochar addition. However, the soil which was amended with B2 and B4 increased nodule numbers to 4.1 ± 1.5 and 3.2 ± 1.0 per plant.

### 2.2. Carbon and Nutrient Concentrations

**Figure 3:**
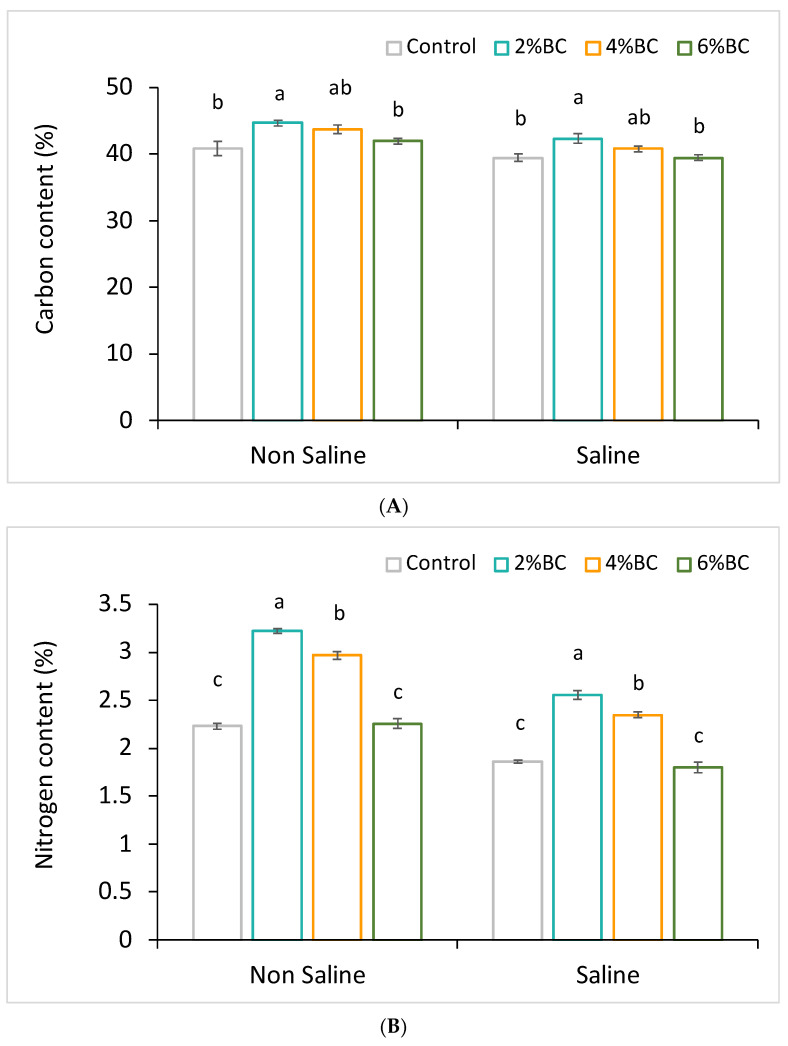

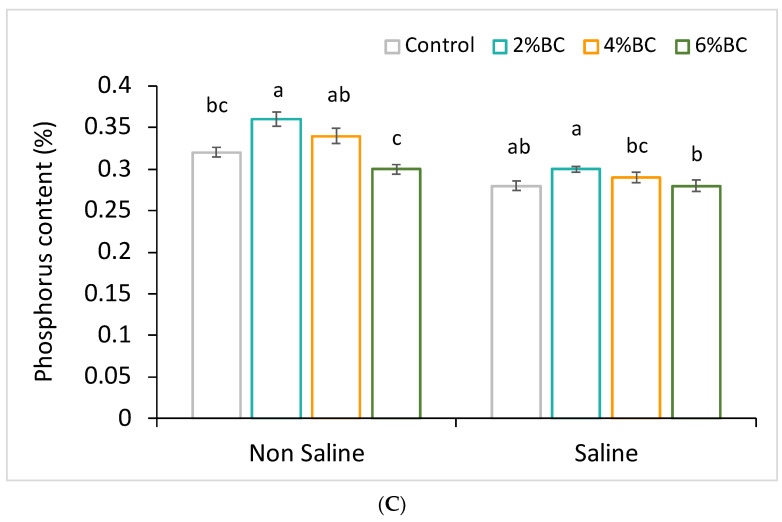
Concentrations (%) of carbon (**A**), nitrogen (**B**), and phosphorus (**C**) in licorice plant tissue grown after application of biochar at 2%, 4%, and 6% concentrations under non-saline and saline soil conditions. The different letters indicate significant differences based on Turkey’s HSD test at *p* < 0.05.

The concentrations of C, N, and P in plant tissue were affected by different biochar concentrations applied under non-saline and saline soil conditions. In the biochar-amended soil, the nutrient concentration in the licorice plant tissues was higher than the plants grown in soil without the addition of biochar. The carbon content in the root tissue of licorice grown in non-saline soil amended with B2 slightly increased by 9% and by 7% under saline soil conditions, respectively (Figure 3A). Significant increases (*p* < 0.05) in N content of plant tissue over the controls were observed after B2 and B4 amendments under non-saline conditions, being 44 and 32% higher, respectively (Figure 3B). Under saline conditions, the soil amended with B2 and B4 showed an increased concentration of plant N content by 37 and 26%, respectively (Figure 3). Among other nutrients analyzed, only the P concentration of plants significantly (*p* < 0.05) increased after the amendment of B2 under non-saline conditions (Figure 3C).

### 2.3. Licorice Root Architecture

**Figure 4:**
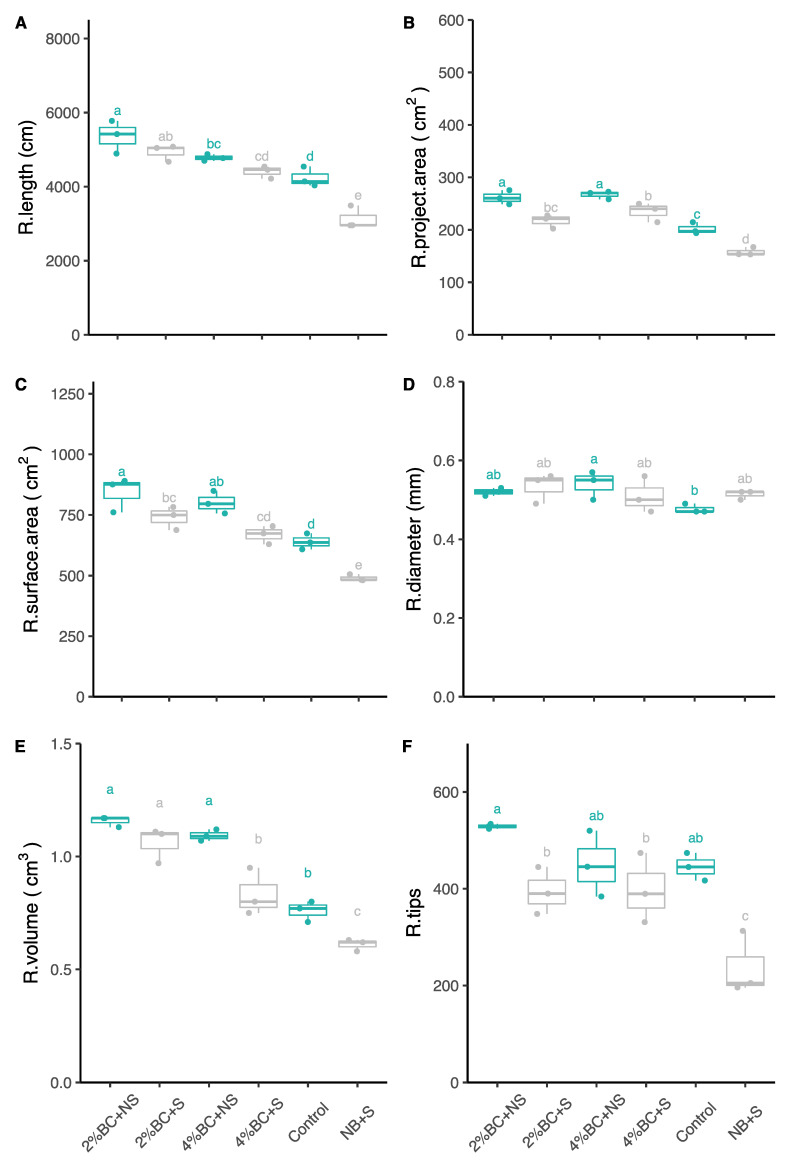
Root morphological traits, i.e., total root length (**A**), project area (**B**), surface area (**C**), root diameter (**D**), root volume (**E**), and root tips (**F**) of licorice grown after application of biochar at 2%, and 4% concentrations under non-saline and saline soil condition. The top and bottom of the box represent 75% and 25% quantile, respectively. The bars of the box represent maximum and minimum values of observations. The line in the box represents the median. The dot represents the value of each individual observation. Letters within each column mark significant differences at *p* < 0.05 based on Duncan’s test. BC: biochar, NB: without biochar, S: saline, NS: non-saline, R.: root.

In general, licorice root architecture showed a differentiated response to different concentrations of biochar amendment. Interestingly, the total root length significantly increased by 26 and 57% under the B2 amendment under non-saline and saline conditions, respectively, as compared to the other treatments (Figure 4A). Root length similarly responded under the amendment of B4. The root length increased at both saline and non-saline soil conditions, although significant differences compared to the control were only observed under saline soil conditions (Figure 4A). Soil amendment changed the root project area, especially when grown under saline conditions (Figure 4B). The root project area significantly increased by 29–32% under non-saline and 37–48% under saline soil conditions compared to the control plant. By comparison, the surface area of the root significantly increased by 31% and 25% under non-saline and 51, and 36% under saline soil conditions compared to the control plants (Figure 4C). As shown in Figure 4D, the root diameter was not affected by biochar additions, except under B4, where an increase of 12% was observed under non-saline soil conditions (Figure 4D). The root volume responded differently to both biochar concentrations and soil conditions. Both B2 and B4 induced up to 52 and 43% higher root volumes under non-saline soil conditions than the control plants (Figure 4E). The root volume responded similarly to the addition of biochar under saline soil conditions, as it was increased by 73% under B2 amendment compared to the control plants (Figure 4E). There was no clear response from the root tips to different biochar additions, as they were significantly (45%) higher after B2 amendment compared to control plants under saline soil conditions only.

### 2.4. Soil Enzymes

**Figure 5:**
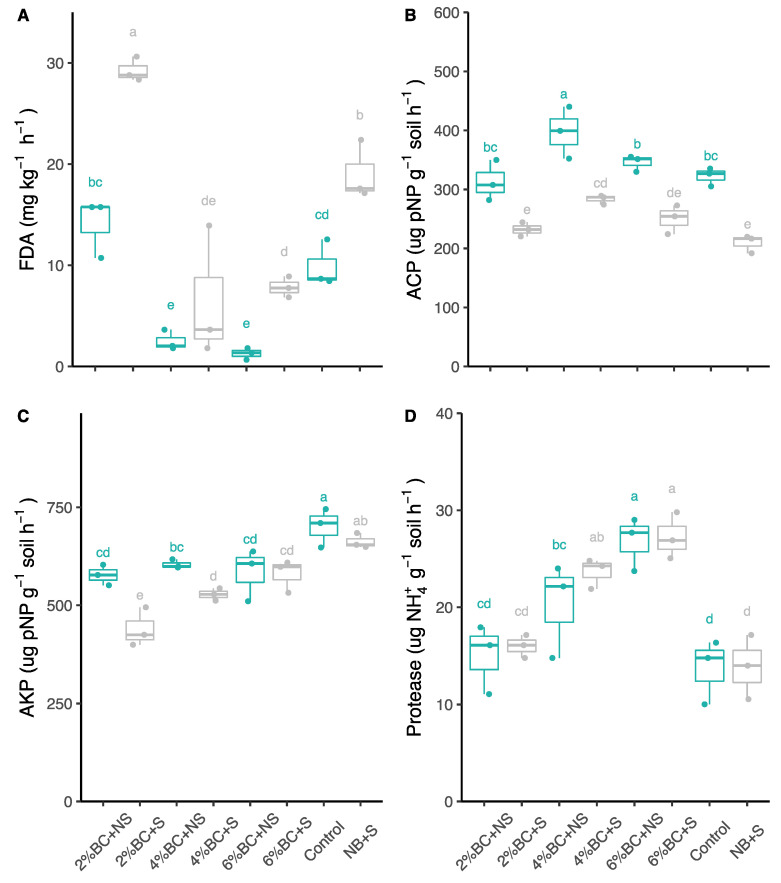
Effect of biochar amendment on FDA hydrolytic activity (**A**), soil acidic phosphomonoesterase activity (**B**), soil alkaline phosphomonoesterase activity (**C**), and soil protease activity (**D**) under non-saline and saline soil conditions. The top and bottom of the box represent 75% and 25% quantiles, respectively. The bars of the box represent maximum and minimum values of observations. The line in the box represents the median. The dot represents the value of each individual observation. Letters within each column mark significant differences at *p* < 0.05 based on Duncan’s test. ACP: acidic phosphomonoesterase activity, AKP: alkaline phosphomonoesterase activity, FDA: fluorescein diacetate hydrolytic activity, Protease: protease activity. BC: biochar, NB: without biochar, S: saline, NS: non-saline.

The biochar amendment of B2 significantly increased fluorescein diacetate (FDA) hydrolase activity in non-saline and saline soil by 45 and 42%, respectively (Figure 5A); however, the activity decreased at the addition of B4 and B6 under both saline and non-saline conditions. The biochar amendments affected the acid and alkaline phosphomonoesterase activities under licorice (Figure 5B). An increase in acid phosphomonoesterase activity by 10 and 25% in non-saline soil and 13 and 32% in saline soil was observed after B2 and B4 amendments compared to the control, respectively. However, the effect was not significant (Figure 5C). In contrast, alkaline phosphomonoesterase activity decreased in soil amended with biochar at all of the applied concentrations (B2, B4, B6). Protease activity significantly increased after biochar additions (Figure 5D), i.e., an increase of 70 and 48% was observed in non-saline and of 96 and 96% in saline soil amended with B4 and B6 compared to the control, respectively (Figure 5D).

### 2.5. Correlations and Redundancy Analysis

**Figure 6:**
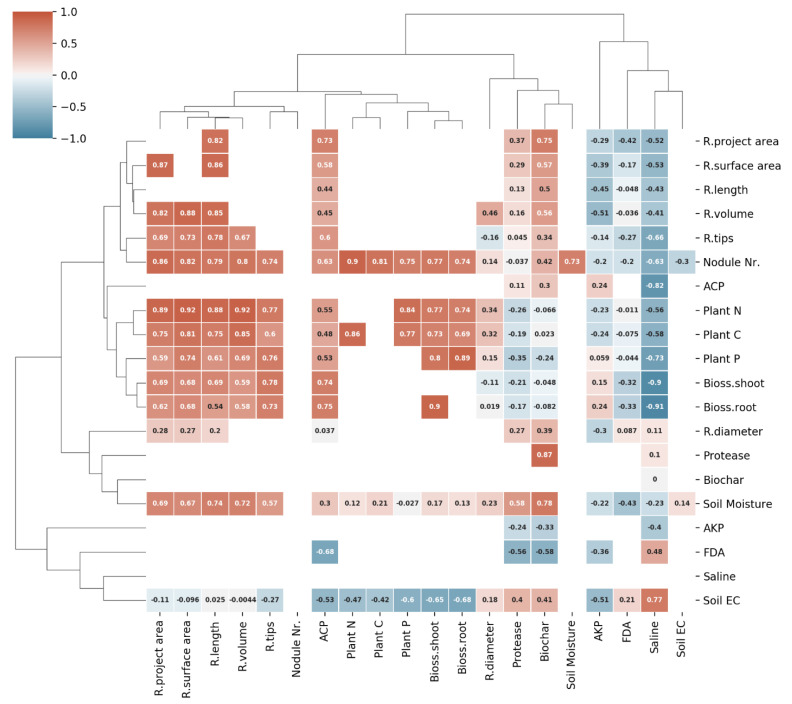
Cluster map of correlations of the root morphological measurements, plant measurements, nodule number, soil properties, saline conditions and biochar additions. Biochar: biochar additions, Bioss.root: root biomass, Bioss.shoot: shoot biomass, EC: soil electric conductivity, Nr.: number, Saline: saline conditions. The colour bar indicates Pearson correlation coefficient. The columns/rows of the data matrix are re-ordered according to the hierarchical clustering result; similar observations are close to each other. The blocks of ‘high’ and ‘low’ values are adjacent in the data matrix.

A cluster map of correlations was plotted to explore the relationship between measurements and retrace cause-effect dependencies, and a RDA was performed. The cluster map of correlations indicates four groups related to root morphological measurements, including root project area, root surface area, and root length. The root volume and tips were clustered with nodule numbers in one group (Figure 6) and significantly and strongly correlated.

**Figure 7:**
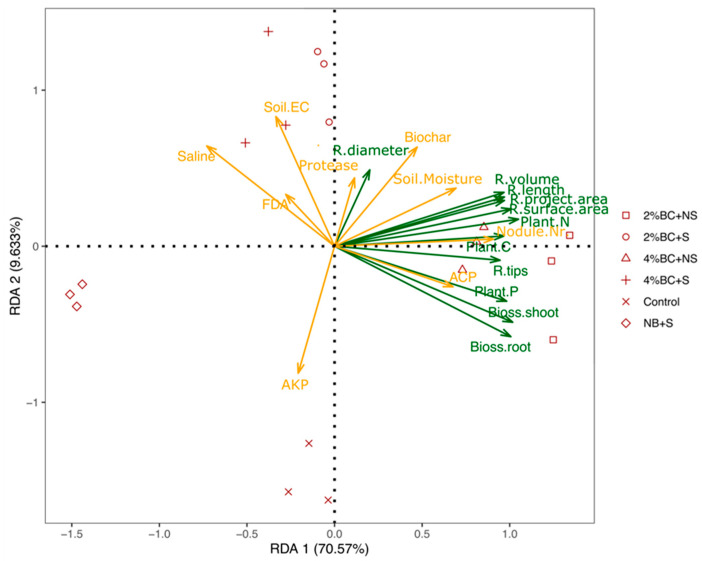
RDA-ordination triplot of variables. Response variables are shown by green arrows; explanatory variables are shown by yellow arrows. The angles between the arrows of the response and explanatory variables indicate correlations. The RDA1 and RDA2 axis explained 70.57% and 9.63% of the total variance, respectively.

Plant tissue measurements, such as contents of N, C, and P, as well as biomass, were clustered with acidic phosphomonoesterase activity in one group. These measurements also significantly and positively correlated with each other. Soil moisture and biochar additions clustered together and showed strong correlations with most of the root morphological measurements apart from root diameter; however, only weak correlations with the plant measurements (Table 1 and Figure 6). Alkaline phosphomonoesterase activity, protease activity, and FDA hydrolytic activity showed weak correlations with root morphological and other plant measurements. Soil EC indicated a weak correlation with root morphological measurements and nodule number and showed a significantly negative correlation with shoot measurements. The saline soil condition indicated a significantly negative correlation with root morphological and aboveground plant parameters. Before the RDA was performed, data of the response variables (root project area, root surface area, root length, root volume, root tips, root diameter, shoot biomass, root biomass, plant N, P and K) as well as for the explanatory variables (biochar addition, saline condition, soil acidic and alkaline phosphomonoesterase activity, protease activity, FDA hydrolytic activity, soil moisture and EC) were normalized. As a result, the first three RDAs explained 70.57%, 9.63%, 2.37% of the total variance (Figure 7).

A permutation test for RDA under a reduced model was performed to test the importance of the chosen model, the axis, and the explanatory variables (Table 2). The significance level of the model of RDA indicates that it can appropriately explain the data (Table 2). The RDA1 axis explained most of the variables and indicated the highest impact. In the explanatory variables, the acidic phosphomonoesterase activity contributes the most to explain the response variables.

The soil EC, alkaline phosphomonoesterase activity and the saline condition also affected the response variables. However, the biochar showed no significant contributions to explain the response variables. Thus, we traced back to the previous results, and a 2% biochar addition showed a positive effect on the root morphological and plant measurements; nevertheless, a 4% or a 6% biochar addition showed no significant impact on plant measurements. The overall contributions of three rates of biochar addition could not be significantly differentiated.

## 3. Discussion

Overall, this study showed that plant growth attributes, root architecture, nutrient acquisition and symbiotic performance of licorice under both non-saline and saline soil conditions improved after the amendment of biochar. However, the concentration of B2 had the most benefits for licorice root and shoot growth, as well as N and P uptake and C allocation under both soil conditions. Biochar dosage could be a crucial factor in determining the biochar potential in improving plant growth [24]. Liu et al. [25] observed a dose-dependent response of rice to biochar related to nutrient availability. They observed an enhancement of rice root grown under 0.05% biochar amendment, but not for 0.1% biochar. In another study, Batool et al. [26] reported improved physiological parameters of *Abelmoschus esculentus* L., such as leaf stomatal conductance and gas exchange rate at 1% biochar application compared 3%. Moreover, growth parameters of licorice were positively affected by 2% biochar treatments under salt stress, indicating alleviation of the adverse impact on plants. Several other reports demonstrated the positive impacts of soil amendments with biochar on plant growth and development under salt stress [27,28]. For example, Farooq et al. [29] observed an increased growth in seedlings and leaf area and reduced oxidative damage and Na+ accumulation in cowpea leaves after biochar application under salt stress conditions. A similar observation was reported for quinoa, where the amendment of biochar significantly increased plant height, shoot biomass, water use efficiency and yield under salt stress compared to control plants [30]. The effects of biochar on plant stress tolerance were also found to be dose-dependent. Ibrahim et al. [23] studied the impact of biochar rates (0%; 2.5%; 5%, 10%) on sorghum growth and physiological attributes under saline conditions. The plant height, leaf area, fresh and dry weight under stress were improved by 2.5 and 5% biochar amendment, whereas a 10% addition of biochar negatively influenced plant physiological properties. The effect has been explained by the regulation of the synthesis of anti-oxidant enzymes in plants induced by biochar [31], and also by a reduction in Na acquisition of plants [32].

The B2 concentration improved the root architecture of licorice under non-saline and saline conditions compared to the other treatments, indicating that a low biochar application rate strongly stimulates root system growth. In addition, the overall contributions of three rates of biochar addition on the root architecture traits were high according to the significant correlation among biochar and root architecture traits from the RDA result. Points for observations of the amendment of B2 and B4 under non-saline conditions can be projected perpendicular on the lines for response and explanatory variables, indicating the response values and explanatory variables at those sites on the triplot. The result also proves the root morphological growth was positively affected by B2 and B4 amendment under non-saline conditions. Similar findings were observed for the halophytes Sesbania (*Sesbania cannabina*) and Seashore mallow (*Kosteletzkya virginica*), where the root system, as well as shoot growth under salt stress, were improved by biochar application [33]. Several reports explain the possible mechanisms leading to the positive effects of biochar on plant growth. Laird et al. [34] observed reduced nutrient leaching and enhanced soil quality after applying biochar. The application rate of B2 increased C, N and P contents in plant tissues under both non-saline and saline conditions. The positive effect of biochar on plant growth was explained by the increased availability of essential nutrients for plant growth and development [35]. The nutrient uptake in plant tissue of corn such as N, P, K, and Mg was stimulated by the biochar amendment of alkaline soil [36]. Biochar is rich in organic carbon and minerals and supplies additional nutrients to the soil available for plant acquisition, improving plant nutritional status and plant development [37]. El-Naggar et al. [38] observed an increased cation exchange capacity (CEC) of soil after biochar amendment, associated with higher nutrient retention. Furthermore, reduced salt stress after biochar application was explained by improving stomatal conductance and water consumption of plants [32,39].

In our study, the nodule numbers decreased in licorice roots grown under saline soil conditions. In an earlier study, Zahran et al. [40] illustrated that abiotic stress might lead to an alteration in the *Rhizobium*-host plant recognition process. Remarkably, application rates of B2 and B4 improved the symbiotic performance of licorice under both non-saline and saline soil conditions. The result of RDA and the cluster map also indicated that biochar positively correlated with nodule numbers. However, only the observations of the B2 and B4 amendment under non-saline conditions can be projected on the line of nodule number on the triplot; apparently, saline conditions offset the effect of biochar to a certain degree. In earlier studies, several reports demonstrated increased nodule numbers in soybean roots after adding biochar [12,41]. Biochar is rich in carbon and several nutrients; some of these nutrients are directly available for microbes. Thus biochar can likely provide sources for bacterial survival and proliferation in soil and rhizosphere [42,43].

Soil enzyme activities responded differently to the biochar concentration applied under both non-saline and saline soil conditions. Fluorescein diacetate (FDA) hydrolase activity increased in soil amended with B2, whereas rates of B4 and B6 additions even inhibited enzyme activity under non-saline and saline conditions. Ma et al. [21] reported that applying 10 t ha^−1^ biochar produced from black cherrywood significantly increased soil FDA hydrolytic activity in a sandy field. Accordingly, an increase in FDA hydrolytic activity, which is an indicator of total microbial activity, was also reported by Chan et al. [15] after adding biochar into the soil. Furthermore, acid phosphomonoesterase activity was stimulated in soil amended with B2 and B4; however, alkaline phosphomonoesterase was inhibited under both soil conditions. In the explanatory variables of the RDA triplot, the acidic phosphomonoesterase activity contributed the most to explaining the response variables. The acidic phosphomonoesterase activity strongly correlated with plant P, shoot dry weight and root dry weight. It is likely that the P uptake may increase organic P mineralization [44] and thus, facilitated the association between plants and microorganisms [45,46]. Furthermore, P-solubilizing microorganisms are promoted to release organic acids to solubilize ortho-P [47,48]. A significant correlation between biochar and acidic phosphomonoesterase activity was also found, suggesting that biochar may have an indirect effect on P cycling for plant growth. Furthermore, soil protease activity strongly increased after applying B2, B4 and B6 under both saline and non-saline conditions. Correspondingly, increased enzyme activities involved in C and N cycles were also detected after adding maize biochar [41]. Thus, the increased enzyme activities in saline-alkaline soils amended with biochar in other studies demonstrated an improved microbial community related to central C, N, and P cycling activities [49,50]. Moreover, urease plays a crucial role in mineralizing soil organic nitrogen, as do phosphatases in transforming soil organic P forms [51,52].

## 4. Materials and Methods

### 4.1. Soil, Biochar and Plant

The soil used in the study was sandy loam, collected from the horizon (0–15 cm depth) of an experimental arable field under irrigation operated by the Experimental Field Station of the Leibniz Centre for Agricultural Landscape Research (ZALF), Müncheberg, Germany. The soil had the following contents: clay and fine silt (7%), coarse and medium silt (19%), sand (74%), C org (0.6%), total N (0.07%), P (0.03%), K (1.25%), and Mg (0.18%), the pH was 6.2. The biochar material was supplied from the Leibniz-Institute for Agrartechnik Potsdam-Bornim e.V. (ATB), Germany. The biochar was produced from maize by heating at 600 °C for 30 min and had the following properties (calculated per dry weight): dry matter (DM% fresh matter)—92.85; ash (%)—18.42; total organic carbon content (%)—75.47, N (%)—1.80; C/N ratio—41.93; Ca (g/kg ^−1^)—9.26; Fe (g/kg^−1^)—11.40; Mg (g/kg^−1^)—4.91; K (g/kg^−1^)—32.26; P (g/kg^−1^)—5.26; pH—9.89; EC—3.08 [53]. Licorice seeds were purchased from an online seed store of Chinese traditional medicine in China and were used for pot experiments.

### 4.2. Plant Growth Experiment

The experiment was conducted in a plant growth chamber at the Leibniz Centre for Agricultural Landscape Research (ZALF). Three concentrations of biochar 2, 4, and 6% (*w*/*w*) were used as a soil amendment. Pots (d = 0.16 m, 3 L) were filled with 1000 g air-dried soil and mixed with crushed chars (particle size < 3 mm). The seeds of licorice (*Glycyrrhiza uralensis* Fisch.) were surface-sterilized using 10% *v/v* NaOCl for 5 min and 70% ethanol for 5 min. After that, seeds were rinsed five times with sterile distilled water and transferred to paper tissue for germination in a dark room at 25 °C for 3–4 days. A total of three seeds were sown to each pot, and after one week, the seedlings were thinned to two plants per pot. The following treatments were set up: (i) plants grown in soil without biochar B0, (ii) plants grown in soil amended with 2% (B2), 4% (B4), and 6% (B6) biochar. Each treatment included four pots and was arranged in a randomized complete block design. The plants were grown under non-saline and saline (50 mM NaCl) conditions for 40 days at a temperature of 24 °C/16 °C (day/night) and in the humidity of 50–60%. Plants were irrigated with tap water containing 50 mM NaCl three times a week. The control treatment was only irrigated by tap water without NaCl. During plant growth, electrical conductivity of soil (EC) and moisture were measured every 3–4 days with UMP-2 BT+ sensor (UGT GmbH, Müncheberg, Germany). Average soil EC and soil moisture during plant growth are presented in Table 1. At harvest, the roots were separated from the shoots, and their biomass was oven-dried at 70 °C for 48 h. The dry weights of root and shoot and the number of nodules were determined from each plant.

### 4.3. Plant and Soil Nutrient Analyses

For the determination of carbon (C), nitrogen (N), and phosphorus (P) concentrations in plant tissues, oven-dried plants were homogenized by milling, and powders of shoots and roots were combined. The powder was analyzed with an inductively coupled plasma optical emission spectrometer (ICP-OES; iCAP 6300 Duo).

### 4.4. Root Morphological and Architectural Traits

Roots were separated from shoots and washed carefully with water. The entire root system was spread outward and analyzed using a scanner system (Expression 4990, Epson, Los Alamitos, CA, USA). Digital images of the root system were analyzed using Win RHIZO software (Régent Instruments, Quebec, Canada) for total root length, root volume, the number of root tips, root surface area, and average root diameter. The total number of nodules per plant root was counted under a stereomicroscope.

### 4.5. Soil Enzyme Measurements

Acid and alkaline phosphomonoesterase activities were assayed, according to Tabatabai and Bremner [54]. The concentration of p-nitrophenol (p-NP) produced in the assays by acid and alkaline phosphomonoesterase activities was calculated from a p-NP calibration curve after subtracting the absorbance of the control at 400 nm wavelength using a Lambda 2 UV-VIS spectrophotometer [55]. Protease activity was assayed using the method described by Ladd and Butler [56]. The ammonium released was calculated by relating the measured absorbance at 690 nm to a calibration graph containing 0, 1.0, 1.5, 2.0, and 2.5 μg of NH_4_^+^-N mL^−1^. The assay of fluorescein diacetate (FDA) hydrolytic activity was performed according to Green et al. [57]. The fluorescein concentration was calculated by reference to a standard curve with 0, 0.001, 0.005, 0.05 and 0.15 mg of fluorescein.

### 4.6. Statistical Analyses

The data were subjected to one-way analysis of variance (ANOVA) using the software package SPSS-22 (SPSS Inc., Chicago, IL, USA). Multiple comparisons of the means were conducted by the least significant difference using Tukey’s Honest Significant Difference (HSD) (LSD) (*p* = 0.05) test. Linear correlation analyses were performed with Pearson’s correlation coefficients to clarify the relationship between various measurements by using python 3.8.1. A cluster map of the correlations was plotted to visualize the results. For further data exploration, a redundancy analysis (RDA) was performed to explain the dependent relationships between the explanatory variables (soil properties) and response variables (plant parameters) using the open-source statistical language R v1.3.1056 (R Studio, Boston, MA, USA). The results of the RDA were plotted on a triplot, on which the angles between the arrows of the response and explanatory variables indicate the correlations.

## 5. Conclusions

The results of our study revealed synergistic effects of concentration-dependent biochar amendments on licorice growth, nutrient uptake, and soil enzyme activities involved in the cycling of C, N and P in sandy loam soil under both non-saline and saline conditions. The improved acquisition of nutrients by licorice was explained by enhanced root growth, bioavailability of nutrients and increased soil microbial activity after biochar amendment. Remarkably, a medium-amount biochar amendment (B2) of the soil mostly improved the root system architecture and thus enabled improved nutrient uptake, and may support nitrogen fixation activity under salt stress. The use of excessive amounts of biochar, however, may result in unfavourable soil physico-chemical or soil ecological conditions such as over-critical aromatic C contents or unfavourable soil aggregation, which may negatively impact plant growth and microbial proliferation.

Overall, our findings underpin the notion of an elaborate interrelationship between biochar concentration and enhanced licorice growth, its root system architecture, symbiotic performance and nutrient acquisition under saline soil conditions.

## Figures and Tables

**Table 1:** Soil electric conductivity (EC) and moisture content during licorice growth in pot experiments.

Condition	Treatment	EC	Moisture
Saline	Control	0.165 ± 0.03	7.5 ± 0.86
	2% BC	0.385 ± 0.08	9.2 ± 0.76
	4% BC	0.421 ± 0.03	9.3 ± 0.95
	6% BC	0.750 ± 0.09	12.0 ± 1.10
Non-saline	Control	0.041 ± 0.01	7.2 ± 0.76
	2% BC	0.062 ± 0.01	11.5 ± 0.50
	4% BC	0.069 ± 0.02	9.5 ± 1.10
	6% BC	0.076 ± 0.01	13.1 ± 0.8

**Table 2:** Permutation test for RDA under reduced model.

		Df	Variance	F	Pr(>F)	Signif.
Model		9	9.8537	5.6962	0.001	***
Axis	RDA1	1	8.0393	41.8259	0.001	***
	RDA2	1	1.0973	5.709	0.219	
	RDA3	1	0.2695	1.4023	0.981	
	RDA4	1	0.215	1.1185	0.987	
	RDA5	1	0.1404	0.7306	0.996	
	RDA6	1	0.038	0.1978	1	
	RDA7	1	0.0303	0.1574	1	
	RDA8	1	0.0204	0.1064	1	
	RDA9	1	0.0035	0.0181	1	
Explanatory variables	Protease	1	0.3749	1.9505	0.158	
	AKP	1	0.8829	4.5933	0.031	*
	ACP	1	4.9067	25.5281	0.001	***
	FDA	1	0.1533	0.7975	0.457	
	Soil.EC	1	1.6688	8.6824	0.002	**
	Soil.Moisture	1	0.5343	2.7798	0.079	.
	Nodule.Nr.	1	0.2909	1.5135	0.22	
	Saline	1	0.8186	4.2591	0.035	*
	Biochar	1	0.2233	1.1617	0.281	
Residual		8	1.5377			

Signif. codes: ‘***’: 0.001, ‘**’: 0.01, ‘*’: 0.05, ‘.’: 0.1.

## Data Availability

Not applicable.

## References

[1] Hayashi H., Hattori S., Inoue K., Sarsenbaev K., Ito M., Honda G. (2003). Field survey of *Glycyrrhiza* plants in Central Asia (1). Characterization of *G. uralensis*, *G. glabra* and the putative intermediate collected in Kazakhstan. Biol. Pharm. Bull..

[2] Patil S.M., Patil M.B., Sapkale G.N. (2009). Antimicrobial activity of *Glycyrrhiza glabra* Linn. Roots. Int. J. Chem Sci..

[3] Sharma V., Agrawal R.C., Pandey S. (2013). Phytochemical screening and determination of anti-bacterial and anti-oxidant potential of Glycyrrhiza glabra root extracts. J. Environ. Res. Devel..

[4] Kushiev H., Noble A.D., Abdullaev I., Toshbekov V. (2005). Remediation of abandoned saline soils using *Glycyrrhiza glabra*: A study for the Hungry steppes of central Asia. Int. J. Agric. Sustain..

[5] Marui A., Nagafuchi T., Shinogi Y., Yasufuku N., Omine K., Kobayashi T., Shinkai A., Tuvshintogtokh I., Mandakh B., Munkhjargal B. (2012). Soil physical properties to grow the wild licorice at semi-arid area in Mongolia. J. Arid. Land. Stud..

[6] Egamberdieva D., Wirth S., Behrendt U., Allah E.F.A., Berg G. (2016). Biochar treatment resulted in a combined effect on soybean growth promotion and a shift in plant growth promoting rhizobacteria. Front. Microbiol..

[7] Mwando E., Angessa T.T., Han Y., Li C. (2020). Salinity tolerance in barley during germination-homologs and potential genes. J. Zhejiang Univ. Sci. B..

[8] Egamberdieva D., Berg G., Lindström K., Räsänen L.A. (2013). Alleviation of salt stress of symbiotic *Galega officinalis* L. (goat’s rue) by co-inoculation of *Rhizobium* with root colonizing *Pseudomonas*. Plant Soil.

[9] Parray J.A., Jan S., Kamili A.N., Qadri R.A., Egamberdieva D., Ahmad P. (2016). Current perspectives on plant growth-promoting rhizobacteria. J. Plant Growth Regul..

[10] Biederman L.A., Harpole W.S. (2013). Biochar and its effects on plant productivity and nutrient cycling: A meta-analysis. GCB Bioenergy.

[11] Saifullah-Saad D., Asif N., Zed R., Ravi N. (2018). Biochar application for the remediation of salt-affected soils: Challenges and opportunities. Sci. Total Environ..

[12] Lehmann J., Rillig M.C., Thies J., Masiello C.A., Hockaday W.C., Crowley D. (2011). Biochar effects on soil biota–a review. Soil Biol. Biochem..

[13] Novak J., Busscher W., Laird D., Ahmedna M., Watts D.W., Niandou M. (2009). Impact of biochar amendment on fertility of a south eastern coastal plain soil. Soil Sci..

[14] Yu O.Y., Raichle B., Sink S. (2013). Impact of biochar on the water holding capacity of loamy sand soil. Int. J. Energy Environ. Eng..

[15] Chan K.Y., Van Zwieten L., Meszaros I., Downie A., Joseph S. (2007). Agronomic values of green waste biochar as a soil amendment. Aust. J. Soil Res..

[16] Manasa M.R.K., Katukuri N.R., Nair S.D., Yang H.J., Yang Z.M., Rong B.G. (2020). Role of biochar and organic substrates in enhancing the functional characteristics and microbial community in a saline soil. J. Environ. Manag..

[17] Tang J.W., Zhang S.D., Zhang X.T., Chen J.H., He X.Y., Zhang Q.Z. (2020). Effects of pyrolysis temperature on soil -plant -microbe responses to *Solidago canadensis* L. derived biochar in coastal saline-alkali soil. Sci. Total. Environ..

[18] Lu H., Li Z., Fu S., Mendez A., Gasco G., Paz-Ferreiro J. (2015). Effect of biochar in cadmium availability and soil biological activity in an anthrosol following acid rain deposition and aging. Water Air Soil Pollut..

[19] Cao Y., Ma Y., Guo D., Wang Q., Wang G. (2017). Chemical properties and microbial responses to biochar and compost amendments in the soil under continuous watermelon cropping. Plant Soil Environ..

[20] Ma H., Egamberdieva D., Wirth S., Li Q., Omari R.A., Hou M., Bellingrath-Kimura S.D. (2019). Effect of biochar and irrigation on the interrelationships among soybean growth, root nodulation, plant P uptake, and soil nutrients in a sandy field. Sustainability.

[21] Ma H., Egamberdieva D., Wirth S., Bellingrath-Kimura S.D. (2019). Effect of biochar and irrigation on soybean-rhizobium symbiotic performance and soil enzymatic activity in field rhizosphere. Agronomy.

[22] Morales M.M., Comerford N., Guirinni I.A., Falkao N.P.S., Reeves J.B. (2013). Sorption and desorption of phosphate on biochar and biochar—Soil mixtures. Soil Use Manag..

[23] Ibrahim H.M., Al-Wabel M.I., Usman A.R.A., Al-Omran A. (2013). Effect of Conocarpus biochar application on the hydraulic properties of a sandy loam soil. Soil Sci..

[24] Gale N.V., Thomas S.C. (2019). Dose-dependence of growth and ecophysiological responses of plants to biochar. Sci. Total Environ..

[25] Liu M., Lin Z., Ke X., Fan X., Joseph S., Taherymoosavi S., Liu X., Bian R., Solaiman Z.M., Li L. (2021). Rice seedling growth promotion by biochar varies with genotypes and application dosages. Front. Plant Sci..

[26] Batool A., Taj S., Rashid A., Khalid A., Qadeer S., Saleem A.R., Ghufran M.A. (2015). Potential of soil amendments (Biochar and Gypsum) in increasing water use efficiency of *Abelmoschus esculentus* L. Moench. Front. Plant Sci..

[27] Hussain M., Farooq M., Nawaz A., Al-Sadi A.M., Solaiman Z.M., Alghamdi S.S., Ammara U., Ok Y.S., Siddique K.H.M. (2017). Biochar for crop production: Potential benefits and risks. J. Soils Sediments.

[28] Egamberdieva D., Ma H., Alimov J., Reckling M., Wirth S., Bellingrath-Kimura S.D. (2020). Response of soybean to hydrochar-based. Microorganisms.

[29] Farooq M., Romdhane L., Rehman A., Al-Alawi A.K.M., Al-Busaidi W.M., Asad S.A., Lee D.J. (2020). Integration of seed priming and biochar application improves drought tolerance in cowpea. J. Plant Growth. Regul..

[30] Yang A., Akhtar S.S., Li L., Fu Q., Li Q., Naeem M.A., He X., Zhang Z., Jacobsen S.E. (2020). Biochar mitigates combined effects of drought and salinity stress in quinoa. Agronomy.

[31] Thomas S.C., Frye S., Gale N., Garmon M., Launchbury R., Machado N. (2013). Biochar mitigates negative effects of salt additions on two herbaceous plant species. J. Environ. Manag..

[32] Akhtar S.S., Andersen M.N., Liu F. (2015). Residual effects of biochar on improving growth, physiology and yield of wheat under salt stress. Agric. Water. Manag..

[33] Zheng H., Wang X., Chen L., Wang Z., Xia Y., Zhang Y., Wang H., Luo X., Xing B. (2017). Enhanced growth of halophyte plants in biochar-amended coastal soil: Roles of nutrient availability and rhizosphere microbial modulation. Plant Cell. Environ..

[34] Laird D.A., Fleming P.D., Wang B., Horton R., Karlen D.L. (2010). Biochar impact on nutrient leaching from a midwestern agricultural soil. Geoderma.

[35] Amini S., Ghadiri H., Chen C., Marschner P. (2016). Salt-affected soils, reclamation, carbon dynamics, and biochar: A review. J. Soils Sediments.

[36] Zhao W., Zhou Q., Tian Z., Cui Y., Liang Y., Wang H. (2020). Apply biochar to ameliorate soda saline-alkali land, improve soil function and increase corn nutrient availability in the Songnen plain. Sci. Total. Environ..

[37] Qayyum M.F., Steffens D., Reisenauer H.P., Schubert S. (2012). Kinetics of carbon mineralisation of biochars compared with wheat straw in three soils. J. Environ. Qual..

[38] El-Naggar A.H., Usman A.R.A., Al-Omran A., Ok Y.S., Ahmad M., Al-Wabel M.I. (2015). Carbon mineralisation and nutrient availability in calcareous sandy soils amended with woody waste biochar. Chemosphere.

[39] Ghezzehei T.A., Sarkhot D.V., Berhe A.A. (2014). Biochar can be used to capture essential nutrients from dairy wastewater and improve soil physico-chemical properties. Solid Earth.

[40] Zahran H.H., Räsänen L.A., Karsisto M., Lindström K. (1994). Alteration of lipopolysaccharide and protein profiles in SDS-PAGE of rhizobia by osmotic and heat stress. World J. Microbiol. Biotech..

[41] Wang X.B., Song D.L., Liang G.Q., Zhang Q., Ai C., Zhou W. (2015). Maise biochar addition rate influences soil enzyme activity and microbial community composition in a fluvo-aquic soil. Appl. Soil. Ecol..

[42] Kolton M., Harel Y.M., Pasternak Z., Graber E.R., Elad Y., Cytryn E. (2011). Impact of biochar application to soil on the root-associated bacterial community structure of fully developed greenhouse pepper plants. Appl. Environ. Microb..

[43] Prendergast-Miller M.T., Duvall M., Sohi S.P. (2011). Localisation of nitrate in the rhizosphere of biochar-amended soils. Soil. Biol. Biochem..

[44] Masto R.E., Kumar S., Rout T.K., Sarkar P., George J., Ram L.C. (2013). Biochar from water hyacinth (Eichornia Crassipes) and its impact on soil biological activity. Catena.

[45] Warnock D.D., Lehmann J., Kuyper T.W., Rillig M.C. (2007). Mycorrhizal responses to biochar in soil-concepts and mechanisms. Plant Soil..

[46] Blackwell M.S.A., Brookes P.C., de la Fuente-Martinez N., Gordon H., Murray P.J., Snars K.E., Williams J.K., Bol R., Haygarth P.M. (2010). Phosphorus Solubilisation and Potential Transfer to Surface Waters from the Soil Microbial Biomass Following Drying–Rewetting and Freezing–Thawing.

[47] Vassilev N., Martos E., Mendes G., Martos V., Vassileva M. (2013). Biochar of Animal Origin: A sustainable solution to the global problem of high-grade rock phosphate scarcity?. J. Sci. Food Agric..

[48] Gul S., Whalen J.K. (2016). Biochemical Cycling of Nitrogen and Phosphorus in Biochar-Amended Soils. Soil Biol. Biochem..

[49] Wu H., Zeng G., Liang J., Chen J., Xu J., Dai J., Li X., Chen M., Xu P., Zhou X. (2016). Responses of bacterial community and functional marker genes of nitrogen cycling to biochar, compost and combined amendments in soil. Appl. Microbiol. Biotechnol..

[50] Shi S., Tian I., Nasir F., Bahadur F., Batool A., Luo S., Yang F., Wang Z., Tian C. (2019). Response of microbial communities and enzyme activities to amendments in saline-alkaline soils. Appl. Soil. Ecol..

[51] Fu Q., Abadie M., Blaud A. (2020). Effects of urease and nitrification inhibitors on soil N, nitrifier abundance and activity in a sandy loam soil. Biol. Fertil. Soils.

[52] Margalef O., Sardans J., Fernández-Martínez M., Molowny-Horas R. (2017). Global patterns of phosphatase activity in natural soils. Sci. Rep..

[53] Reibe K., Götz K.P.C.L., Ross T.F., Doering F., Ellmer L., Ruess R. (2015). Impact of quality and quantity of biochar and hydrochar on soil collembola and growth of spring wheat. Soil. Biol. Biochem..

[54] Tabatabai M.A., Bremner J.M. (1969). Use of p-nitrophenol phosphate for the assay of soil phosphatase activity. Soil Biol. Bioch..

[55] Acosta-Martinez V., Mikha M.M., Sistani K.R., Stahlman P.W., Benjamin J.G., Vigil M.F., Erickson R. (2011). Multi-location study of soil enzyme activities as affected by types and rates of manure application and tillage practices. Agriculture.

[56] Ladd J.N., Butler J.H.A. (1972). Short-term assays of soil proteolytic enzyme activities using proteins and dipeptide derivatives as substrates. Soil. Biol. Biochem..

[57] Green V.S., Stott D.E., Diack M. (2006). Assay for fluorescein diacetate hydrolytic activity: Optimalization for soil samples. Soil Biol. Biochem..

